# miRNA-Based Diagnosis of Schizophrenia Using Machine Learning

**DOI:** 10.3390/ijms26052280

**Published:** 2025-03-04

**Authors:** Vishrut Heda, Saanvi Dogra, Valentina L. Kouznetsova, Alex Kumar, Santosh Kesari, Igor F. Tsigelny

**Affiliations:** 1 Scholars Program, CureScience Institute, San Diego, CA 92121, USA; hedavishrut@gmail.com; 2 MAP Program, University of California San Diego, La Jolla, CA 92093, USA; saanvid8@gmail.com; 3 San Diego Supercomputer Center, University of California San Diego, La Jolla, CA 92093, USA; vkouznet@ucsd.edu; 4 Department of Sciences, CureScience Institute, San Diego, CA 92121, USA; 5 Computing and Mathematical Sciences Department, California Institute of Technology, Pasadena, CA 91125, USA; alex.k.kumar@gmail.com; 6 Department of Neuro-Oncology, Pacific Neuroscience Institute, Santa Monica, CA 90404, USA; santosh.kesari@providence.org; 7 Department of Neurosciences, University of California San Diego, La Jolla, CA 92093, USA

**Keywords:** miRNA, machine learning, schizophrenia, diagnostics

## Abstract

Diagnostic practices for schizophrenia are unreliable due to the lack of a stable biomarker. However, machine learning holds promise in aiding in the diagnosis of schizophrenia and other neurological disorders. Dysregulated miRNAs were extracted from public sources. Datasets of miRNAs selected from the literature and random miRNAs with designated gene targets along with related pathways were assigned as descriptors of machine-learning models. These data were preprocessed and classified using WEKA and TensorFlow, and several classifiers were tested to train the model. The Sequential neural network developed by authors performed the best of the classifiers tested, achieving an accuracy of 94.32%. Naïve Bayes was the next best model, with an accuracy of 72.23%. MLP achieved an accuracy of 65.91%, followed by Hoeffding tree with an accuracy of 64.77%, Random tree with an accuracy of 63.64%, Random forest, which achieved an accuracy of 61.36%, and lastly ADABoostM1, which achieved an accuracy of 53.41%. The Sequential neural network and Naïve Bayes classifier were tested to validate the model as they achieved the highest accuracy. Naïve Bayes achieved a validation accuracy of 72.22%, whereas the sequential neural network achieved an accuracy of 88.88%. Our results demonstrate the practicality of machine learning in psychiatric diagnosis. Dysregulated miRNA combined with machine learning can serve as a diagnostic aid to physicians for schizophrenia and potentially other neurological disorders as well.

## 1. Introduction

Current diagnostic practices in psychiatry are based on clinical evaluation of the patient’s symptoms and duration of illness, often as per the *Diagnostic and Statistical Manual of Mental Disorders, Fifth Edition* (DSM-V) and the International Classification of Diseases (ICD-11) [1,2,3]. Affecting ~1% of the global population [4], schizophrenia (SCZ) is a mental disorder that is exhibited in the form of positive symptoms (delusions, hallucinations) and negative symptoms (avolition, asociality) [5]. Presently, there are no stable and dependable biomarkers for SCZ, making diagnosis difficult as it relies entirely on a psychiatrist’s analysis, which is challenging in the early stages [6]. Diagnosis is initiated with the questioning of patients to assess criteria and symptom severity using the Positive and Negative Syndrome Scale (PANSS) [7], a process noted to be subjective and variable [8], followed by trial-and-error treatments [9,10]. Furthermore, neurological disorders such as SCZ, bipolar disorder, autism, and other psychiatric disorders have been suggested as being part of a spectrum of increasing severity, suggesting that many neurological disorders are related to one another and have similar etiopathogenesis. This often leads to misdiagnoses because their respective diagnostic categories are poorly defined within the DSM/ICD manuals (symptoms defined using unspecific umbrella terms), and the symptoms are highly similar [5,9,11,12]. Thus, searching for an objective diagnostic aid or method is critical in psychiatric research, particularly for SCZ.

Machine learning (ML) has presented itself in computational psychiatry as a potentially helpful diagnostic aid [9]. ML can focus on individual prediction, which is better suited to psychiatry than group-level analysis (as used in classical statistical methods) and can deal with observational data acquired from real-life settings, such as neuroimaging data (electroencephalography (EEG) and magnetic resonance imaging (MRI) scans) and blood/metabolic samples (peripheral blood plasma, serum, etc.), which may have incomplete or missing information [9]. Particularly, ML has shown great value in analyzing large quantities of neuroimaging data (namely EEG, MRI, and magnetoencephalography (MEG) data) to differentiate between healthy controls and SCZ [13,14].

EEG and MEG ML models, such as those by Shim et al. [15] and Kim et al. [16], employ a three-step process of feature extraction, feature selection, and classification. Nodes from EEG/MEG scans are characterized by source/sensor level features trimmed and classified using ML to create the models [15]. MRI-based ML models are another popular diagnostic tool for consideration and are applicable for both structural MRI (sMRI) and functional MRI (fMRI) [17]. Network analysis was conducted by Jo et al. [18], where brain networks were graphed using neural elements and synaptic connections represented by nodes and edges, respectively. The graph features from the network properties were used for classification, with two different types of properties: nodal and global. Diagnosing speech abnormalities in SCZ using ML was explored by Espinola et al. [19]; vocal acoustic analysis was conducted by extracting features from patient audio files and applying classifiers.

Search for SCZ, blood-based biomarkers in previous studies have shown that gene expression analysis using ML holds potential for diagnostics [20,21,22]. The existing literature refers to diverse potential biomarkers (e.g., proteomics, metabolomics) [23] for SCZ. Our focus was on genomics, specifically miRNA, as no diagnostic tool based on these currently exists. Studies exploring blood-based biomarkers have found specific genes to be dysregulated and models have been built based on mRNA [24,25,26,27]. However, to the extent of our knowledge, no work has been carried out developing ML models based on miRNA as diagnostic tools for SCZ [22,28,29,30,31].

miRNAs have been studied and are compelling potential biomarkers for other neurological disorders. Xu et al. [21] used dysregulated miRNAs for the diagnosis of Alzheimer’s disease (AD) and built an ML model as a diagnostic tool for AD. Studies applying miRNA and ML in classifying other neurological disorders, such as Parkinson’s disease and dementia, have also shown high performance [32,33].

## 2. Results

### 2.1. The Models’ Accuracy Depends on the Number of Correctly Classified Instances out of the Total

**Figure 1:**
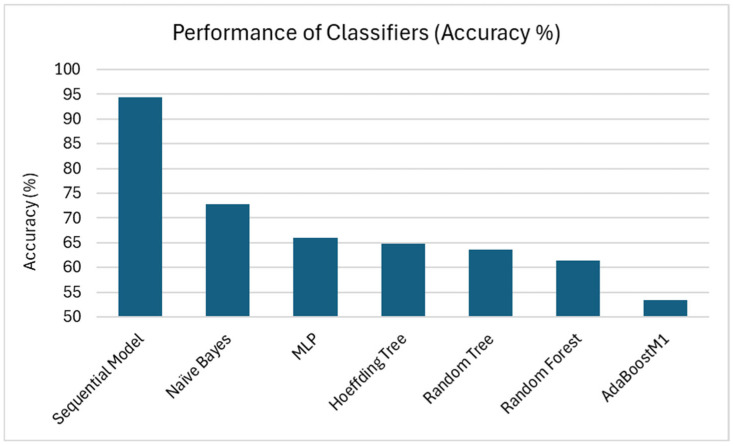
Performance of Classifiers by Accuracy (%). The accuracy was calculated by dividing the number of correctly classified instances by the number of total samples and multiplying by 100. The bars represent the accuracy of each classifier, with the sequential model performing the best.

Of the seven classifiers we tested, ADABoostM1 performed the worst, with an accuracy of 53.41%, with 47/88 miRNA correctly classified as being SCZ-associated or randomly selected; followed by Random forest with an accuracy of 61.36% (54/88 correctly classified instances); and Random tree with an accuracy of 63.64% (56/88 correctly classified instances); then Hoeffding tree with an accuracy of 64.77% (57/88 correctly classified instances), the multilayer perceptron (MLP) with an accuracy of 65.91% (58/88 correctly classified); and Naïve Bayes with an accuracy of 72.73% (64/88 correctly classified instances). The model that performed the best while training with 10-fold cross-validation was the deep-learning sequential model, with an accuracy of 94.32% and 83/88 miRNA correctly classified. The performance results are presented in Figure 1.

**Figure 2:**
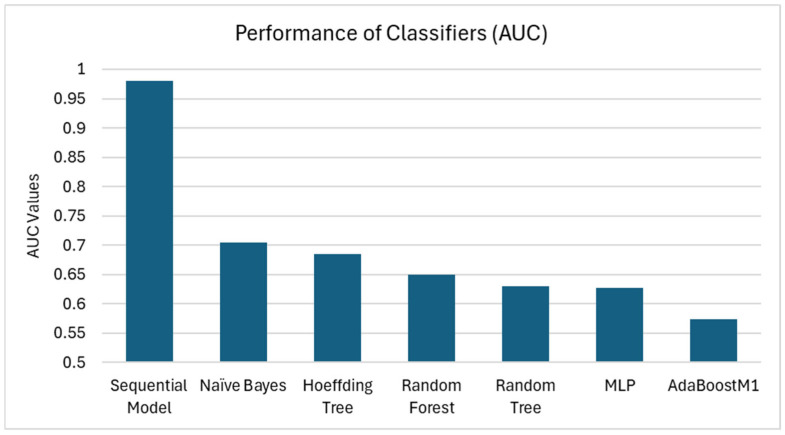
Performance of Classifiers estimated by AUC. The bars represent each classifier’s individual AUC (area under receiver operating characteristic (ROC) curve). The higher the AUC, the more accurately the model classified the instances. Similar to Figure 1, the Sequential model performed the best.

**Figure 3:**
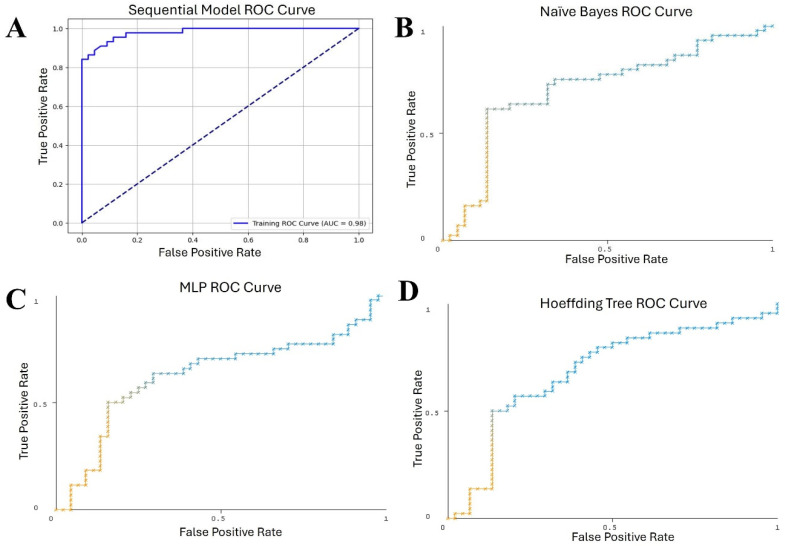
ROC curves for best-performed classifiers. (**A**) Sequential model classifier. (**B**) Naïve Bayes classifier. (**C**) MLP classifier. (**D**) Hoeffding tree classifier. The *x* axis represents the false positive rate (how many cases were incorrectly classified as positive) and the *y* axis represents the true positive rate (how many cases were correctly classified as positive). The area under the curve (AUC) measures the classifier’s performance; the higher the AUC, the more instances were correctly classified and the better the classifier. The color represents the threshold value set for the pair Color–Threshold of true False positive rate–True positive rate point.

Another metric we used to understand the performance was the average AUC (area under the receiver operating characteristic (ROC) curve). The ROC curve plotted the true positive rate (TPR) against the false positive rate (FPR), with the area under the graph (AUC) used as an accurate measure of each classifier’s performance. The greater the AUC, the better the classifier performed, signifying a higher TPR and/or a lower FPR. In this context, the AUC values for the classifiers we tested were as follows: AdaBoostM1 had the lowest AUC of 0.578, followed by MLP (AUC of 0.627), Random forest (AUC of 0.636), and Random tree (AUC of 0.650), followed by Hoeffding tree (AUC of 0.685), Naïve Bayes (AUC of 0.705), and finally, the Sequential model (AUC of 0.98). Figure 2 plots the AUC values for all explored classifiers and Figure 3 illustrates the ROC curves for the four best-performing classifiers: the Sequential model, Naïve Bayes, MLP, and Hoeffding tree.

In the final step, which was the validation of the model, the model’s accuracy was measured by the number of instances in the independent test in the ‘selected’ class and how many instances out of the total were in the ‘random’ class. The top two trained models were the sequential neural network and the Naïve Bayes classifier. Since both obtained relatively high accuracies when being trained, they were selected to be used to validate the results. The new independent validation set was inputted into both of these classifiers and their performances were evaluated. The sequential model performed better, with a validation accuracy of 88.88%, whereas the model trained using Naïve Bayes had a validation accuracy of 72.22%.

### 2.2. KEGG Pathway Analysis of miRNA and Gene Targets

Upon building the descriptors file for inputting to WEKA, we found a list of gene targets for each miRNA. When attribute selection was performed in WEKA, the following 26 features were selected by WEKA for training the model: *CLIP1*, *DSCAM*, *MAPK10*, *ERCC2*, *E2F3*, *MAP3K9*, *TBC1D16*, *ASB5*, number of hydrogen bonds, hepatitis B pathway, Chagas disease pathway, mean mass, AACA motif, AAUC motif, ACUA motif, AGAG motif, AGUU motif, CAGU motif, CGGU motif, GAUG motif, GCAA motif, GUGU motif, GUUA motif, UCGU motif, UCUU motif, and UGGC motif. Of the selected features, eight were gene targets (*CLIP1*, *DSCAM*, *MAPK10*, *ERCC2*, *E2F3*, *MAP3K9*, *TBC1D16*, *ASB5*). These gene targets were highly valuable for correctly classifying the miRNA, meaning they were profoundly regulatory in SCZ etiopathogenesis. However, this list of targets was small, so, a list of genes commonly expressed with our original list from WEKA was obtained. GeneFriends is an online gene database built via transcriptome sequencing, which can identify genes coexpressed with each other through coexpression networks to aid in interpreting gene function and regulation [34,35]. Applying GeneFriends’ analysis to our list from WEKA granted us a larger list of genes, which we ran through DAVID to find the KEGG pathways implicated in SCZ [36,37]. These DAVID pathways connected our miRNAs to their gene targets according to the list of genes elucidated by attribute selection in WEKA and genes added by GeneFriends, thereby elucidating their role in SCZ etiopathogenesis [38,39,40]. Below, we describe several pathways elucidated by the DAVID program.Neuroactive ligand–receptor interaction (hsa04080) is one of these pathways. It controls G protein-coupled receptors (GPCRs) for dopamine and serotonin and is directly related to neuropathology and neuroplasticity, known to be common factors making an individual more prone to SCZ [41,42].

The glutamatergic synapse pathway (hsa04724) is also implicated. Glutamate is a neurotransmitter that has a role in the cerebral cortex and limbic system, brain regions implicated in the onset of SCZ [43]. Drugs correcting irregular cortico-limbic glutamatergic neurotransmission have shown promise for reducing the negative and cognitive symptoms of SCZ [44]. Dysfunction of cortico-limbic glutamatergic neurotransmission causes cognitive impairments, and negative symptoms likely contribute to the manifestation of SCZ [44,45].

Long-term depression (hsa04730) was the next pathway suggested by the DAVID program to be involved in SCZ. Many proteins that play a role in long-term depression have been found to be correlated with SCZ risk [46]. Furthermore, pathways involved in depression are also involved in SCZ [47]. Schijven et al. [48] reported that pathways representing synaptic signaling processes (a process noted to be dysfunctional in SCZ), such as hsa04730 (long-term depression) and hsa04724 (glutamatergic synapse), were found to play a role in SCZ.

The synaptic vesicle cycle pathway (hsa04721) controls the release of neurotransmitters and synaptic plasticity, both of which processes are known to be disrupted in SCZ [49,50], and various neurological disorders can result from synaptic cell cycle dysfunction [51]. Many proteins that make up the synaptic vesicle, such as synaptophysin, synaptobrevin, and syntaxin-1, have been discovered to be irregularly expressed in various brain regions [49,50]. The mutated gene *CLTCL1*, reported to be associated with SCZ, plays a role in synaptic plasticity, a process regulated by hsa04721 (the synaptic vesicle cycle pathway), thereby indicating that pathway’s involvement in SCZ [52].

SCZ has been associated with an upregulated haptoglobin *Hp2* gene. Its precursor is zonulin, which affects intercellular tight junction integrity [53]. The tight junction pathway (hsa04530) is involved in processes decisive to neurodevelopment and synaptic function [54]. The tight junction pathway overlaps heavily with another pathway relating to cell adhesion molecules, and both are critical to synaptic formation and neurotransmission at glutamatergic and GABAergic synapses [55], which have been noted to be dysfunctional in SCZ [56]. Additionally, many genes overlapping in tight junction and similar pathways (i.e., cell adhesion molecules, SNARE) have been implicated in increased SCZ risk [54].

These genes that are known to be associated with SCZ progression are important in the model’s decision regarding whether a given miRNA could be a biomarker for schizophrenia, indicating the classifier’s reliability. The model bases its decisions on existing biological trends and the known effects of miRNAs, since miRNAs that target these genes are more likely to be classified as SCZ biomarkers.

## 3. Discussion

We explored SCZ diagnosis methods described in open sources and found that these consisted primarily of various neuroimaging techniques such as MRI [17,57,58,59,60], EEG [15], and MEG [16]. Scientists have explored potential biomarkers for SCZ (genomics/proteomics, metabolomics) [23]. Both sets of methods are effective diagnostic approaches, as SCZ is influenced by genetics and environmental risk factors [61,62,63,64]. Furthermore, SCZ manifests itself with detectable brain abnormalities [15]. SCZ has a strong genetic influence, with a high heritability rate of 80–85% [65,66], making genetic markers and methods of diagnosis based on genetics highly effective. miRNA has been proven to be expressed differently in SCZ patients in the comparison of healthy control and SCZ patient peripheral blood samples. Scientists have found several individual miRNAs that are dysregulated in SCZ patients [29,67]. However, this data had not been applied to construct a diagnostic tool. Applying ML presents a solution to this problem, as it makes it possible to use pattern recognition approaches such as classical statistical methods and can also identify distinctions in data that classical statistical methods cannot. AI preprocessing, classification, clustering, and the attribute selection abilities of ML, as well as its easy operation (since, in many cases, it does not require high-level knowledge of statistics or programming) [9], make it reliable and effective.

Neuroimaging is effective, as seen with several MRI-data-based diagnostic tools that have achieved accuracies of 90%+ [68,69,70]. However, nearly all the neuroimaging models constructed in the studies applying neuroimaging to SCZ diagnosis have used patients in the severe and later stages of SCZ (with highly evident symptoms) to train the model [6,15,17,60], using Leave-one-out-cross-validation (LOOCV) to assess separate data from patients in the severe stages of SCZ [6,15,17,60]. In these later stages, brain and brain function abnormalities are relatively easier to detect than in the earlier stages of SCZ onset, rendering neuroimaging models less viable in the early stages. In contrast, miRNA-based diagnostic tools are just as effective in the early stages of SCZ onset as they are reliant on the patient’s genetics, which remains unaltered regardless of the patient’s condition. miRNA-based diagnostic tools were demonstrated to be almost as effective, as we achieved an accuracy of 94.32% with our model, and these can be used for initial detection, which other methods are currently unable to do.

The data used in this research had a much better classification rate when tested with the Sequential neural network and Naïve Bayes model than the other classifiers. These trends may be due to the inherent nature of the data. Naïve Bayes classifiers operate under the assumption that features are conditionally independent, which is beneficial when features are loosely correlated or for data without clear trends [71]. Sequential models successfully capture complex non-linear relationships through detailed architectures and multiple layers that can interpret detailed patterns [72]. In contrast, decision-tree-based models such as random forest and Hoeffding tree operate by sectioning certain features and, therefore, struggle to deal with non-linear or minimal data requiring deeper feature interactions [73].

The classifier developed in this research can be implemented in a clinical setting by first identifying miRNAs that are dysregulated in the serum of the peripheral blood of a patient. Then, the described procedure can be applied to collect the sequence descriptors, target genes, and predicted pathways of that miRNA. These data should be input into the machine learning classifier to predict whether the miRNA is associated with schizophrenia, for the clinician to see. If a clinical patient has a high number of symptoms of SCZ-associated miRNA, they can be monitored, and more in-depth diagnosis techniques can be used to determine whether they have this neurological disorder and possible methods to treat it. This model is meant to be used in the initial stages of SCZ detection to identify the disorder quickly through miRNA profiling. The genes and pathways identified as important features for the model’s decision-making could also be explored as potential therapeutic targets in the future. The model can be generalized to a wide range of demographics, as the miRNA used to train the model was taken from diverse patients and was consistently present across a wide range of independent studies.

However, miRNA-based diagnostic models also encounter problems; genetic factors are not the only thing to affect the development and onset of SCZ, which combines genetic and environmental factors [61,62,63,64]. Thus, genetic markers alone cannot detect SCZ proficiently in clinical settings. Furthermore, our model was built using limited data from the existing literature and could be far more accurate if constructed using data from more patients. Also, the model does not consider variability in miRNA between the different forms or stages of schizophrenia, due to the lack of data associating biomarkers with specific stages and phases of disease development. More nuanced models can be created once more biomarkers are identified to be specific to certain stages. Furthermore, some studies from which the miRNAs for training were identified may not have accounted for changes in miRNA expression due to forms of antipsychotic medicine. However, the model can still be used for initial screening before neuroimaging, as it identifies fundamental trends in miRNA expression and has high accuracy. miRNAs are effective long-term biomarkers of schizophrenia that demonstrate strong potential for early diagnosis of this disease through machine learning [74].

## 4. Methods and Materials

**Figure 4:**
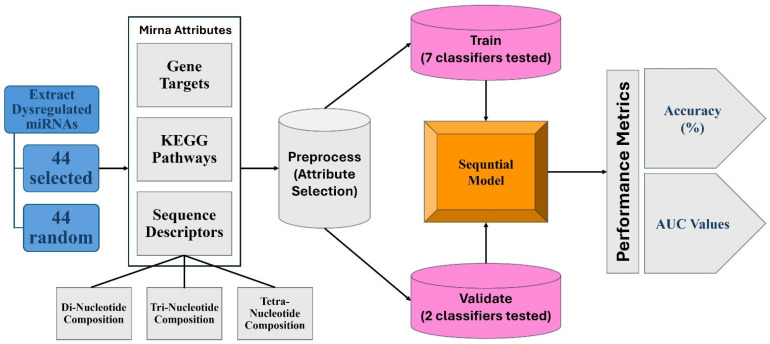
Model’s Architecture. Diagram of methods displaying miRNA acquisition, attribute designation, model development, and performance measurement.

We built and validated ML models based on previously discovered dysregulated miRNAs from open sources [29,31,67,75]. Model’s Architecture is presented in Figure 4. The software and databases used included WEKA, version 3.9.6 (Waikato Environment for Knowledge Analysis, University of Waikato, Hamilton, New Zealand), the ML software used for classification and pattern recognition [76]; TensorFlow, version 2.16, Google Brain, San Diego, CA, USA), a software library specifically for deep learning development [77]; miRDB, version 6.0, (University of Chicago, IL, USA), an online microRNA target prediction database, [78,79]; the DAVID, version v2024q2 (Database for Annotation, Visualization, and Integrated Discovery, Laboratory of Human Retrovirology and Immunoinformatics, Frederick National Laboratory for Cancer Research, Frederick, MD, USA), bioinformatics resource, a functional annotation and microarray analysis tool [36,37]; and miRBase, release 22.1 (University of Manchester, Manchester, MCR, UK), a database/archive for miRNA sequences and annotations [80,81,82,83,84,85].

We initiated the study by identifying dysregulated peripheral blood-based miRNAs associated with SCZ, from open sources (with *p*-values less than 0.05). These miRNAs were consistently dysregulated across many SCZ patients and implicated in SCZ processes by multiple independent studies, indicating that the dysregulation was not caused by external factors such as stress or diet. Studies were specifically chosen if they were conducted on various genders, races, and demographics, to ensure the generalizability of the trained model. miRNA from patients with other neurological disorders in addition to SCZ were disregarded. We also used random miRNAs unrelated to SCZ to serve as a control group for the ML model. This procured a total of 88 miRNAs (44 selected and 44 random) from either serum or peripheral blood. Next, we elucidated their gene targets from the microRNA target prediction database miRDB (mirdb.org. accessed 29 August 2024) [78,79], where we selected the potential target genes for all 88 miRNAs. Only target genes that received a prediction accuracy score of 97–100% from the software (high likelihood of being a gene target of the given miRNA) were selected. In addition, the target genes for each miRNA (from miRDB [79]) were designated KEGG, version 112.0 (Kyoto Encyclopedia of Genes and Genomes; Kanehisa Laboratories, Bioinformatics Center, Institute for Chemical Research, Kyoto University, Uji, Kyoto, Japan) pathways from DAVID (david.ncifcrf.gov, accessed 14 September 2024) [36,37]. Only the pathways that the DAVID software assigned as being significant (*p* < 0.05) were chosen [36,37].

A Python, release 3.13.0 (Python Software Foundation, Beaverton, OR, USA), script was then developed to process the miRNA information and the associated gene targets. The script collected data from miRBase [80,81,82,83,84,85], miRDB [78,79], and DAVID [36,37], integrating gene target information and pathway annotations for each miRNA. The script calculated a set of sequence descriptors for each miRNA, including features such as nucleotide composition, sequence length, and other relevant sequence properties. Finally, the script transformed the gene target data and sequence descriptors into a one-hot-encoded dataset. This data was used to train and validate the ML model with the WEKA software [76]. WEKA [76] was first used to perform attribute selection using the subset evaluation, and only the 26 most important features were chosen. The software then allowed us to create a model (diagnostic tool) to predict whether a given miRNA was SCZ-associated or randomly selected, using data extracted from miRDB [78,79] and DAVID [36,37]. Specifically, classifiers such as Hoeffding tree were trained and tested with 10-fold cross-validation, which iterated the data multiple times and used different subsections for training and testing each time. TensorFlow [77] was also used to create a sequential neural network with five layers: a 100-neuron dense input layer with ReLU activation, three 70-neuron, 50-neuron, and 30-neuron dense ReLU hidden layers, and, finally, the sigmoid activation output. To validate the model, we found another set of 18 miRNAs that were aberrantly expressed in SCZ patients, identified from open sources [86,87,88,89,90,91]. The validation set was constructed following the same attributes, with the appropriate sequence descriptor data of the training set and a “yes” or “no” to indicate the presence of the selected gene targets or KEGG pathways for each miRNA. Any miRNAs already used to train the model were disregarded when selecting the validation set. The validation set was inputted into the two best-performing trained models (the two classifiers with the highest accuracy and AUC on the training set), using the WEKA software [76] and TensorFlow [77] to evaluate performance on unseen data.

Seven ML algorithms or classifiers were tested for their potential to classify schizophrenia: ADABoostM1, Random forest (RF), Random tree (RT), Multilayer perceptron (MLP), Naïve Bayes (NB), Hoeffding tree (HT), and Sequential neural network. ADABoostM1 is a boosting algorithm that runs a weak learning algorithm repeatedly, adjusting the distribution each time based on the error of the classifier’s hypothesis. The weak learning algorithm calls on multiple classifiers over a specific number of iterations (thereby combining multiple classifiers). Then, it aborts the loop once the number of iterations has been completed [92]. RT is a decision tree where predictions are made like a flow chart, starting from a root node, branching into further decisions and several ‘child nodes’ [22] to maximize heterogeneity, making predictions (choosing variables) that best allow the root node to split into distinct groups. If the samples within a node are part of the same group, “then that node becomes a leaf node” [22]. RF is an algorithm for classification and regression tasks that combines these weak classifiers or decision trees to create a forest, in a method known as ‘bagging’ [73]. In RF, an ensemble of decision trees is generated via selection carried out by random vectors over a distribution held constant for all trees. These trees then vote for the most popular class, making up the random forest classifier [93]. The Naïve Bayes classifier depends on simplifying assumptions and probabilistic semantics to classify data [71]. It “assumes that the predictive attributes are conditionally independent given the class” and “that no hidden or latent attributes influence the prediction process” [71]. Bayes’ rule is used to estimate the probability of each class and determine the model’s accuracy based on a vector of observed attribute values [71]. Multilayer perceptrons are among the best-known and most-used types of neural networks, in which learning is based on the minimization of errors between the desired output and the neural output [94]. For this, backpropagation through the network is used for algorithm learning [94]. In backpropagation, gradient descent optimizes weights by propagating errors backward [94]. MLPs have multiple layers, of which not all are input/output layers; some help to make decisions and are known as ‘hidden’ or intermediate layers [94]. An HT is an efficient decision tree mining algorithm based on the VFDT (very fast decision tree) model [95]. HTs are unique as they are designed for time-changing data streams and use the Hoeffding bound to govern their decision-making when splitting nodes [96]. The Hoeffding bound is a statistical test that helps to decide the best attribute when a node in a decision tree is split [95]. It is helpful because unlike the previous classifiers mentioned, it does not rely on the common and generalizing assumption that the distribution of values is equal, and the bound holds true regardless of the distribution [96]. Sequential models are deep learning neural networks trained through a series of layers stacked in a linear sequence, with each neuron in one layer connected to the next layer. The ReLU (rectified linear unit) makes the output equal to the input if it is positive, speeding up training. In contrast, the sigmoid activation function is typically used at the end of the model to map the input to 0 or 1 in binary classification. These models typically handle one-directional data and understand more complex relationships between attributes [72].

The study’s critical part was establishing the genes associated with each miRNA using a miRNA target database. miRDB [78,79] is an online database cataloging miRNAs from several species, with 7086 mature miRNAs (3,519,884 gene targets) for all species and 2656 mature miRNAs (1,610,510 gene targets) for homo sapiens [78]. miRNAs control gene targets and thus control various biological processes and pathways. miRDB has a nomenclature system reliant on mature miRNAs to organize data systematically (this reduces redundancy, so that miRNAs are not confused with their precursors in functional annotation). It has a functional annotation tool that allows new information to be constantly updated either via manual entry or input from automated bioinformatics pipelines [78]. The other key feature of miRDB (in addition to functional annotation) is its ability to predict gene targets based on its unique algorithm [78]. This is how we concluded the effects of miRNA on SCZ etiology based on the genes/targets associated with miRNA and, thus, the biological processes/pathways associated with each miRNA.

DAVID is a database that can elucidate signaling pathways using gene lists. DAVID performs enrichment analysis to obtain the ‘enriched annotation terms’, or the biological processes associated with each gene [36,79].

## 5. Conclusions

This study centered around creating a diagnostic tool that applied known dysregulated miRNAs (shown to have potential as biomarkers) extracted from literature with ML software. A tool was created to differentiate SCZ-associated miRNA biomarkers from randomly selected ones. The model achieved a peak accuracy of 94.32% with the sequential classifier and, after validation, reached an accuracy of 88.88% on an independent test set. This demonstrated that miRNA with ML can be applied in clinical practice to identify biomarkers that can serve as diagnostic aids for physicians to distinguish schizophrenia patients from healthy controls. However, our model still needs considerable work, and one direction to explore would be to attempt to combine multiple methods of diagnosis. When combining features of fMRI with those of sMRI, the models performed better at differentiating between SCZ patients and healthy controls [97,98]. The same occurred with EEG models when combining source and sensor features [15]; it appears that having multiple and diverse inputs improved the performance of the models. This can be explored with our model by combining various input sources, such as other genetic and metabolomic markers, as a prospective direction for future studies.

## Data Availability

Data is contained within the article.
